# PANET: A GPU-Based Tool for Fast Parallel Analysis of Robustness Dynamics and Feed-Forward/Feedback Loop Structures in Large-Scale Biological Networks

**DOI:** 10.1371/journal.pone.0103010

**Published:** 2014-07-24

**Authors:** Hung-Cuong Trinh, Duc-Hau Le, Yung-Keun Kwon

**Affiliations:** 1 School of Electrical Engineering, University of Ulsan, Ulsan, Republic of Korea; 2 School of Computer Science and Engineering, Water Resources University, Hanoi, Vietnam; Universitat Pompeu Fabra, Barcelona Research Park of Biomedicine (PRBB), Spain

## Abstract

It has been a challenge in systems biology to unravel relationships between structural properties and dynamic behaviors of biological networks. A Cytoscape plugin named NetDS was recently proposed to analyze the robustness-related dynamics and feed-forward/feedback loop structures of biological networks. Despite such a useful function, limitations on the network size that can be analyzed exist due to high computational costs. In addition, the plugin cannot verify an intrinsic property which can be induced by an observed result because it has no function to simulate the observation on a large number of random networks. To overcome these limitations, we have developed a novel software tool, PANET. First, the time-consuming parts of NetDS were redesigned to be processed in parallel using the OpenCL library. This approach utilizes the full computing power of multi-core central processing units and graphics processing units. Eventually, this made it possible to investigate a large-scale network such as a human signaling network with 1,609 nodes and 5,063 links. We also developed a new function to perform a batch-mode simulation where it generates a lot of random networks and conducts robustness calculations and feed-forward/feedback loop examinations of them. This helps us to determine if the findings in real biological networks are valid in arbitrary random networks or not. We tested our plugin in two case studies based on two large-scale signaling networks and found interesting results regarding relationships between coherently coupled feed-forward/feedback loops and robustness. In addition, we verified whether or not those findings are consistently conserved in random networks through batch-mode simulations. Taken together, our plugin is expected to effectively investigate various relationships between dynamics and structural properties in large-scale networks. Our software tool, user manual and example datasets are freely available at http://panet-csc.sourceforge.net/.

## Introduction

The dynamical behavior of a biological network is highly related to its structural characteristics [1], [2]. In particular, there have been many studies of the effects of feedback loops (FBLs) and feed-forward loops (FFLs) on network robustness [3]–[5]. For example, networks robustly converging to a fixed state are inclined to have a larger number of positive FBLs and a smaller number of negative FBLs [3]. Coherent coupling of FBLs is a design principle of a robust cell signaling network [6]. It was also shown that the number of FBLs involving a node is positively correlated with the functional importance of the node [7], and two diseases are more likely to be comorbid if the genes associated with each disease are connected with FBLs of a relatively short length in a human signaling network [8]. With respect to a feed-forward loop structure, its dynamical role was also explained in various biological processes, for example, in guaranteeing robust carbohydrate uptake in *Escherichia coli* [4] or adapting to variations in the critical morphogen level in a switch of the cell fate [9]. In addition, the degree to which an FFL consisting of three positive transcriptional regulators was sensitive to primary level perturbation was related to the robustness [10], and the coherent FFLs can be considered as a design principle of human signaling networks that improve network robustness against update-rule perturbations [5].

Inspired by those studies, NetDS, a Cytoscape [11] plugin, was recently developed to analyze the robustness-related dynamics and FFL/FBL structures of networks [12]. However, there were some significant limitations with regard to fully utilizing the plugin. First, analyses of large-scale networks were limited due to high computational complexity (Actually, the plugin was effective for networks having about several tens of nodes). In addition, the observed result could not be validated as a design principle because there was no function to simulate the result on a large number of arbitrary random networks. To resolve these limitations, we developed a new software tool by making the core algorithms run in parallel and providing a new function of a batch-mode simulation on a large number of random Boolean networks (RBNs). For the parallel computation, we employ an OpenCL parallel computing platform, which is an open-source library designed to run on any modern central processing units (CPUs) or graphics processing units (GPUs) (see Text S1 in File S1 for a brief introduction to OpenCL). Thus, our new plugin PANET can be used on any computer equipped with multi-core CPUs and/or GPUs that can support the OpenCL library for analyses of network robustness and FBL/FFL structures. For the batch-mode simulation, PANET first generates a large number of RBNs from various models, and examines their robustness-related dynamics and FBLs/FFLs structures during batch processing. This new function helps us to conclude whether a finding in real biological networks can be a design principle or not by examining the consistency of the finding in random networks. We tested PANET in two case studies based on two large real networks. In the first case study, we found that coherent FFLs are frequently found in the real signaling networks and explained that abundant coherent FFLs are needed to improve robustness against update-rule perturbations. In the second case study, we observed that coherent FBLs are ubiquitously found in the real signaling networks but there is no significant relation between coherent couplings of FBLs and robustness against initial-state perturbations unlike the previous studies having shown the positive correlation of them in very small networks.

## Material and Methods

In this section, we explain the implementation issues of PANET. We first provide a summary of a Boolean network model which was used for robustness calculation in PANET. Next, we describe the parallelization of two core algorithms: robustness computation and FBLs/FFLs detection. Finally, we explain a function for a batch-mode investigation on RBNs.

### A Boolean network model and robustness-related dynamics

To compute the robustness of a network, we employed a Boolean network model [7], [13], [14]. A Boolean network is represented with a directed graph *G*(*V*, *A*), where *V* = {*ν*_0_, *ν*_1_, …, *ν_N_*_-1_} is a set of Boolean variables and *A* is a set of ordered pairs of Boolean variables called directed links. Each *ν_i_*∈*V* has a value of 1 (“on”) or 0 (“off”), which represents the possible states of the corresponding elements. In gene networks, value 1 represents the “turn-on” status in which a gene is expressed. The state of a Boolean network is defined as a vector of the states of all nodes. A directed link (*ν_i_*, *ν_j_*) has a positive (“activating”) or negative (“inhibiting”) relationship from *ν_i_* to *ν_j_*. The value of each variable *ν_i_* at time *t*+1 is determined by the values of *k_i_* other variables 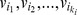 with a link to *ν_i_* at time *t* by the Boolean function 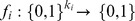, and all variables are also synchronously updated. Hence, the update rule can be written as the following formula: 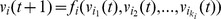. In many previous studies, biological networks such as signaling pathways and gene-regulatory networks were successfully described with Boolean network models that employ conjunction or disjunction update-rules [15]–[18].

A state of *G* is defined as a vector of values *ν*_0_ through *ν_N_*_-1_. A state trajectory starts from an initial state and eventually converges to either a fixed-point or limit-cycle attractor. These attractors can represent diverse biological network behaviors such as multi-stability, homeostasis, and oscillation [19]–[21]. For each attractor, the basin of an attractor can be defined as a set of initial states that will eventually converge to the attractor. The size of a basin indicates the ratio of the number of initial states belonging to the basin to the entire number of initial states. Based on the definition of the attractor, we can introduce the notion of robustness in terms of converging dynamics. If a network sustains the converged attractor against perturbations that affect some nodes, it is robust against those perturbations. Therefore, the change in the converging attractor can be interpreted as a loss of robustness. This concept has been widely used in a number of previous studies [22]–[26]. Here, we considered two types of perturbations: an initial-state perturbation and an update-rule perturbation. Given a sequence of update-rules *f* = [*f*_0_, *f*_1_, …, *f_N_*_-1_] and an initial state *s = *[*ν*_0_(0), *ν*_1_(0), …, *ν_N_*_-1_(0)], an initial-state perturbation at a node *ν_i_*∈*V* is a situation in which *s* is changed to *s*′* = *[*ν*_0_(0), …, 1-*ν_i_*(0),…, *ν_N_*_-1_(0)], *i.e.*, the corresponding initial value is switched to 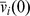 (the negation of *ν_i_*(0)). An initial-state perturbation represents the abnormal (or malfunctioning) status of a protein or gene caused by a mutation. On the other hand, an update-rule perturbation at a node *ν_i_*∈*V* involves a scenario where *f* is changed to *f*′ = {*f*_0_,…, *f_i_*_,_′, …, *f_N_*_-1_}, where *f_i_*′ is the disjunction rule if *f_i_* is the conjunction rule and vice versa. The update-rule perturbation may represent a change in the relationship between nodes. For a set of considered initial states *S*, we now define the robustness of a node *ν_i_* against the initial-state perturbation and the update-rule perturbation, denoted as *γ_s_*(*ν_i_*) and *γ_r_*(*ν_i_*), respectively, as follows:
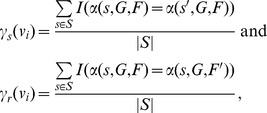
where *α*(*s*, *G*, *F*) represents the attractor that *s* will converge to in a Boolean network *G* specified by the set of update-rules *F*, and *I*(*condition*) denotes an indicator function that returns 1 if the *condition* is true and 0 otherwise. In other words, *γ_s_*(*ν_i_*) and *γ_r_*(*ν_i_*) represent the probability with which a network sustains the converging attractor against initial-state and update-rule perturbations, respectively. Furthermore, the robustness of a network *G* against the initial-state perturbation and the update-rule perturbation, denoted as *γ_s_*(*G*) and *γ_r_*(*G*), respectively, are defined as follows:
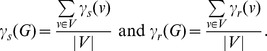



In other words, the robustness of a network is computed by averaging the robustness of all nodes in the network.

### Parallel computation of robustness-related dynamics

**Figure 1:**
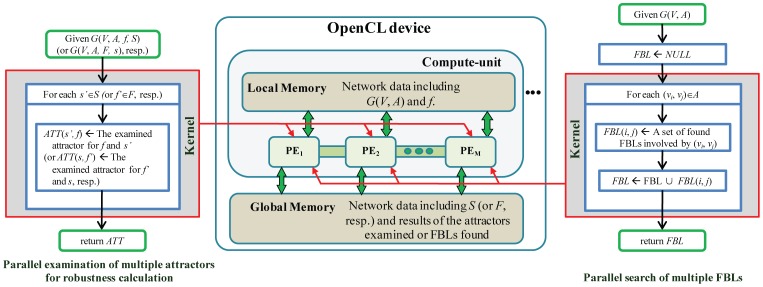
Core OpenCL-based implementation for robustness calculation (left) and FBLs examination (right) in parallel. It is shown how in parallel PANET computes converging attractors over all possible initial-states or all update-rules (left) and searches FBLs (right).

Unfortunately, it is very time-consuming to compute converging attractors over all possible initial-states or all sequences of update-rules, which is a required process when computing robustness. To reduce the computation time, we introduced a parallel algorithm which utilizes numerous processing units in CPUs or GPUs (see Text S2 in File S1 for the pseudo-code). More specifically, a parallel algorithm is needed to compute multiple attractors over a lot of different initial-states (or update-rules, respectively) for the robustness calculation. This task is parallelized in PANET by assigning the attractor computation for each initial-state (or each sequence of update-rules, respectively) to a Processing Element (PE) of a CPU/GPU (see the left part in Figure 1).

### Definition of feedback and feed-forward loops

A feedback loop, which can be described as a circular chain of relationships, is very common and plays an important role in the dynamic behaviors of cellular signaling networks [2], [27], [28]. Given a network, a feedback loop is a closed simple cycle in which all nodes, with the exception of the starting and ending nodes, are not revisited. More specifically, *ν*_0_ → *ν*_1_ → *ν*_2_ → … → *ν_L_*_-1_ → *ν_L_* is an FBL of length *L*(≥1) if there are links from *ν_i-_*_1_ to *ν_i_* (*i* = 1, 2, …, *L*) with *ν*_0_ = *ν_L_* and *ν_j_* ≠ *ν_k_* for *j*, *k*∈{0, 1, …, *L*-1}. The number of FBLs of node *ν* denotes the number of different FBLs starting from *ν*. In addition, the sign of an FBL is easily determined by the parity of the number of negative relationships involved. If the parity number is even or zero, the FBL is positive; otherwise, it is negative. We further consider the notion of coupled FBLs because of their relationship to the dynamic properties of a network [29], [30]. To define the coupling of feedback loops, let us first define a sub-sequence of a feedback loop. Given a feedback loop *P* = *ν*_0_ → *ν*_1_ → *ν*_2_ → ··· → *ν_L_*, we call *u*_0_ → *u*_1_ → ··· → *u_M_* a sub-sequence of *P* of length *M* if there is *i*∈{0, …, *L*} such that *ν*_(*i*+*j*)%(*L*+1)_ = *u_j_* for *j* = 0, …, *M*. If there is a non-empty common sub-sequence between two feedback loops, there is a coupling between the feedback loops. When the two FBLs involved in the coupling have the same sign, it is a coherent coupling; otherwise, it is an incoherent coupling. In addition, we define the intersection length of a coupling as the number of common links.

A feed-forward loop is also an important motif in network dynamics [31]–[33]. Thus, we implemented a feed-forward loop based on the following definition. Given a network *G*(*V*, *A*), *ν*_0_ → *ν*_1_ → *ν*_2_ → … → *ν_L_*_-1_ → *ν_L_* is a simple path of length *L*(≥1) if there are links from *ν_i-_*_1_ to *ν_i_* (*i* = 1, 2, …, *L*) with *ν_j_* ≠ *ν_k_* for *j*, *k*∈{0, 1, …, *L*}. Similar to the definition of the sign of an FBL, the sign of a simple path is determined by the parity of the number of negative links involved. If the parity number is even or zero, the path is positive; otherwise, it is negative. When a pair consisting of a source node (*ν_i_*) and a sink node (*ν_j_*) has two or more simple paths, the set of simple paths is a feed-forward loop starting from *ν_i_* and ending at *ν_j_*. Furthermore, an FFL is coherent if all simple paths involved have the same sign; otherwise, it is incoherent.

### Parallel detection of FBLs and FFLs

In NetDS, a user could search FBLs and FFLs for a specified maximal length (*L*). This function was implemented using a depth-first search, which is a kind of graph traversal method. It will take a long time for a large network to be traversed, though, so we introduced a parallel algorithm for FBL and FFL searches to reduce the computation time by using the OpenCL library (see Text S3 in File S1 for the pseudo-code).

More specifically, each directed link should be examined to determine whether there exist FBLs/FFLs involving the link or not. This task is parallelized in PANET by assigning the examination of each link to a PE of a CPU/GPU (see the right part of Figure 1). In addition, we improved the search speed by avoiding redundant examination (see Text S3 in File S1).

### A batch-mode simulation on random Boolean networks

**Figure 2:**
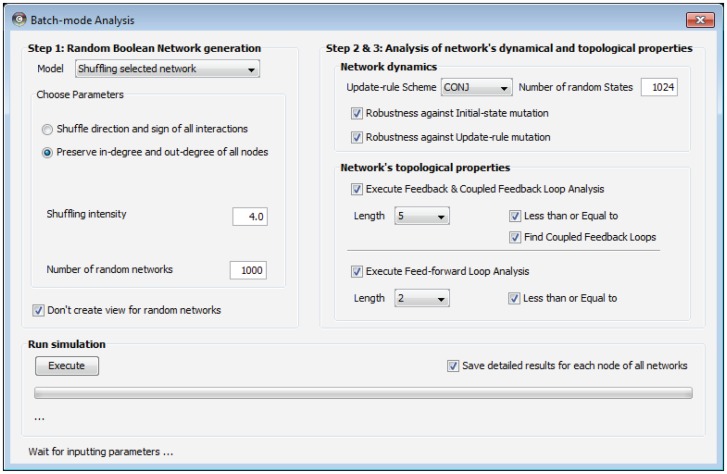
User interface for a batch-mode simulation on RBNs. There are three steps for configuring parameters of the batch-mode simulation: selecting an RBN generation model, setting the number of considered initial-states and the type of update-rule schemes, and specifying the maximal length of FBLs/FFLs to be searched.

We developed a function for a batch-mode simulation on RBNs to examine if a finding in biological networks holds in RBNs or not. As shown in Figure 2, the batch-mode simulation requires three steps for configuring parameters. The first step is to select an RBN generation model from among five models: Barabási-Albert (BA) model [34], Erdős-Rényi (ER) model [35], an Erdős-Rényi variant model [12] and two shuffling models. Actually, all of them have been widely used to investigate biological networks [3], [5]–[8], [12], [36]–[39]. The BA model uses a preferential attachment scheme, which is a type of network growth model, as follows. The desirable number of nodes (*N*), the number of nodes of a seed network (*e*), and the number of interactions that should be added at each iteration (*d*) are given as parameters. A small seed network *G*(*V*, *A*) is then created, where *V* = {*ν*_1_, *ν*_2_, …, *ν_e_*} and *A* = {(*ν_i_*, *ν_j_*) | *i*, *j* = 1,2, …, *e*, *i*≠*j*}, i.e., a complete network. At each iteration, a new node *ν* is added to *V*. Then, *d* different interactions that individually connect *ν* and *ν*′∈*V* \{*ν*} are newly added to *A*, where *ν*′ is determined with a probability proportional to the connectivity of *ν*′ (the connectivity of a node is defined as the number of interactions incident to the node), and both the direction and sign of the added interactions are specified uniformly at random. This iteration process is repeated until |*V*| = *N*. In the ER model, the desirable number of nodes (*N*) and a probability (*p*) are given as parameters. The decision whether to create an interaction from an arbitrary node *ν* to another arbitrary node *ν*′ is then independently determined with a probability *p.* PANET also uses a variant of the ER model where the desirable numbers of nodes (*N*) and interactions (*E*) are given as parameters. An RBN is then generated in such a way that *E* different interactions are chosen uniformly at random out of *N*×(*N*-1) possible candidates. Moreover, we implemented two shuffling techniques where a reference network should be given. The first shuffling technique creates random networks by shuffling the direction and the sign of every interaction from the reference network (Shuffle I). More specifically, each directed link denoted by (*ν_i_*, *ν_j_*, *τ*) where *ν_i_*, *ν_j_*, and *τ* denote a starting node, an ending node, and the sign of the link, respectively, is replaced by one of (*ν_i_*, *ν_j_*, *τ*), (*ν_i_*, *ν_j_*, *-τ*), (*ν_j_*, *ν_i_*, *τ*), and (*ν_j_*, *ν_i_*, *-τ*) uniformly at random [5]. On the other hand, the other shuffling technique creates random networks by rewiring the edges of the reference network such that the in-degree and the out-degree of all nodes are conserved (Shuffle II) [36], [37]. More specifically, a pair of directed links (*ν_a_*, *ν_b_*, *τ_ab_*) and (*ν_c_*, *ν_d_*, *τ_cd_*) such that there is no link from *ν_a_* to *ν_d_* and from *ν_c_* to *ν_b_* is randomly selected, and the pair is replaced by a new pair of links (*ν_a_*, *ν_d_*, *τ_ab_*) and (*ν_c_*, *ν_b_*, *τ_cd_*). In our tool, the number of rewirings is set to the multiplication of the value of the "Shuffling intensity" parameter and the number of edges of the reference network. We note that the shuffling models generate RBNs whose structure is more similar to the reference network than BA, ER, and ER-variant models because the degree distribution is conserved.

The second step of parameter configuration is to set the number of considered initial-states and the type of update-rule schemes (see the subsection “A Boolean network model and robustness-related dynamics”). We provide three update-rule schemes; CONJ-DISJ, CONJ, and DISJ. CONJ and DISJ denote that each node of an RBN would be assigned a conjunction and disjunction function, respectively. CONJ-DISJ denotes that each node of an RBN would be assigned a conjunction or disjunction function randomly. The final step is to specify the maximal length of the FBLs/FFLs to be searched. By clicking the ‘Execute’ button, the tool examines robustness and FFL/FBL structures of the generated RBNs in batch mode. After it is completed, all the analyzed results are saved in the two resulting files: “net_based_result.txt” and “node_based_result.txt”. The former and the latter describe the network-based and the node-based results, respectively (see Text S4 in File S1 for the format of the output file). We note that parallel computation during robustness calculation and FBLs/FFLs search made it possible to massively analyze RBNs of the same size with real signaling networks in a practical period of time.

## Results

In this section, we first present scalability of PANET by running it with a large-scale human signaling network (HSN) with 1,609 nodes and 5,063 links [40]. Then, we show two case studies regarding the relationships between dynamics and structural properties in two signaling networks, HSN and a canonical cell signaling network (CCSN) obtained from http://stke.sciencemag.org/, including 818 nodes and 1,801 links [6]. In particular, we tried to verify the relationships through the batch-mode simulation on a lot of large-scale RBNs in those case studies. We note that it could be conducted in a practical time by the parallel implementation of main functions in PANET.

### Scalability by parallel computation in PANET

To show the scalability of PANET over the original plugin NetDS, we compared their running times for calculating robustness and for searching FBLs in the HSN. All were tested on a system with an NVIDIA GeForce GTX 680 GPU with 1536 processor at 1 GHz, four-core Intel Core2 Quad Q9400 CPU 2.66 GHz, and 8 GB of memory. Table 1 shows the result. In the table, “PANET on CPU” and “PANET on GPU” represent results of PANET executed on CPU and GPU only, respectively. Longer FBLs or increases in the number of considered initial-states result in greater performance improvement. More specifically, the maximum speedup factor of the FBLs searching task was 165 with a maximal length of 7. In the robustness calculation, the speedup factor was 453 when the number of considered random initial-states was 1,000. Therefore, the analyses of robustness and FBLs/FFLs could be conducted in a practical time in PANET. In addition to the parallel implementation, the performance improvement of PANET is higher than expectation considering the specification of the used CPU and GPU. This was achieved through utilization of an efficient memory type and a simple data type besides the parallelization. More specifically, the network data can be stored in the local cache memory which is the fastest memory type by the OpenCL library in PANET whereas it was stored in the normal memory in NetDS. In addition, we modified the ‘String’ class-based implementation in NetDS to a primitive data type (the signed integer) based implementation in PANET for fast processing.

**Table 1:** Performance comparisons of PANET with NetDS.

(a) Running time (seconds) for FBLs search					
Maximal Length (*L*)	NetDS (A)	PANET on CPU (B)	Speedup (A/B)	PANET on GPU (C)	Speedup (A/C)
4	1	1	1	1	1
5	7	2	4	1	7
6	72	3	24	3	24
7	1,984	12	165	21	94

(b) Running time (seconds) for robustness calculation					
Number of considered initial-states (*S*)	NetDS (A)	PANET on CPU (B)	Speedup (A/B)	PANET on GPU (C)	Speedup (A/C)
50	4,016	19	211	11	365
100	8,026	41	196	19	422
150	12,069	58	208	30	402
200	16,220	78	208	37	438
1000	80,600	387	208	178	453

Another point to be noted is that the performance improvement of “PANET on GPU” over that of “PANET on CPU” is not so large. Basically, GPU processes “single instruction & multiple data (SIMD)” whereas CPU does “multiple instruction & multiple data (MIMD)”. Therefore, the execution time on GPU is highly affected by how many diverging branch codes are involved in the execution path. The tasks such as FBLs/FFLs search and robustness calculation necessarily include many branching statements and this might reduce the utilization efficiency of multiprocessors in the GPU.

### Case study 1: Relationship between coherent feed-forward loops and network robustness

Some previous studies have shown that an individual coherent structure of the FFL can play an important role in the dynamic behavior of biological networks. For example, a coherent FFL serves as a sign-sensitive delay element in transcription networks [41], and prolongs flagella expression in *E. coli* [42]. In addition, there was a study showing that coherent FFLs are abundant in biological networks and these coherent FFLs can improve network robustness against update-rule perturbations [5]. In particular, they found that coherent FFLs increase robustness because these structures induce downstream nodes to be robust against update-rule perturbations. However, the effect of coherent FFLs on the robustness in a large-scale network was not proven. For this reason, we further conducted two batch-mode simulations on RBNs to examine whether such a coherent FFL structure is a design principle or not.

**Figure 3:**
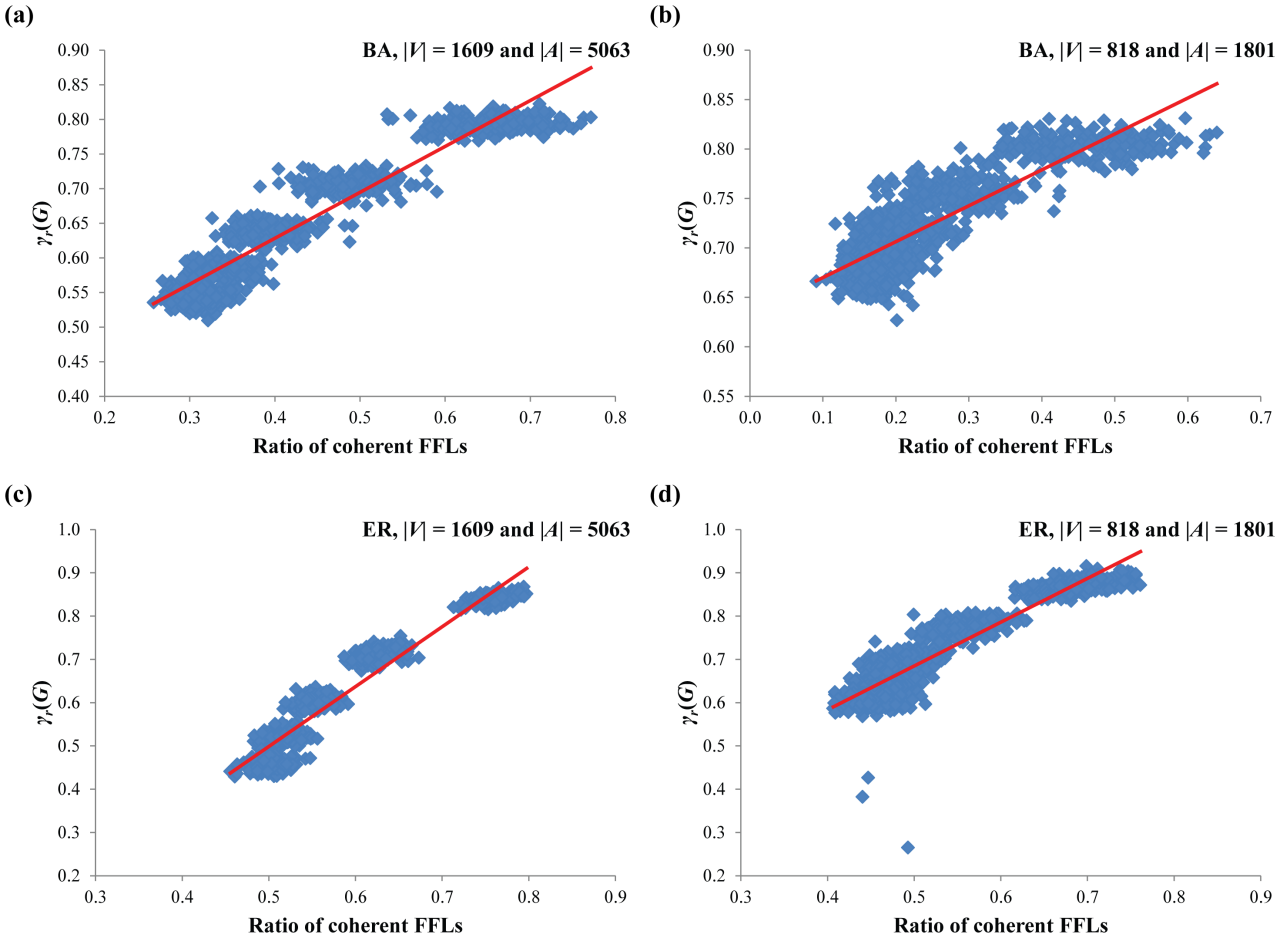
Relationship between the ratio of coherent FFLs and update-rule robustness in large-scale Boolean networks by BA and ER models. (**a**) Result of BA-based RBNs of the same size with HSN. (**b**) Result of BA-based RBNs of the same size with CCSN. (**c**) Result of ER-based RBNs of the same size with HSN. (**d**) Result of ER-based RBNs of the same size with CCSN. The maximal length of examined FFLs is set to 4 or 6 for (a) and (c), or (b) and (d), respectively. For robustness against update-rule perturbation, |*S*| is set to 1,024. The slopes of the regression lines are 0.66315, 0.36297, 1.38296 and 1.01162 in (a), (b), (c) and (d), respectively (all P-values<0.0001 using t-test).

In the first simulation, we examined whether coherent FFLs are ubiquitously found in HSN and CCSN. To this end, we generated eight sets of 1,000 random Boolean networks of the same size with each of the real network by using Shuffle I, Shuffle II, BA and ER models, respectively, and examined the ratio of coherent FFLs within them. As shown in Table 2, we found that the ratio of coherent FFLs in the real networks is significantly greater than those of the random Boolean networks (using one-sample t-test, all P-values<0.0001) except for ER model cases. This indicates that coherent FFLs are likely to be ubiquitously found in the large-scale signaling networks. In the second simulation, we examined whether the highly coupled structure of FFLs also has an effect on the network robustness or not as in the previous study [5]. To this end, we generated eight sets of 1,000 random Boolean networks of the same size with the HSN (|*V*| = 1,609 and |*A*| = 5,063) and the CCSN (|*V*| = 818 and |*A*| = 1,801), respectively, by using BA, ER, Shuffle I and Shuffle II models and examined the relation of the ratio of coupled FFLs on the robustness against the update-rule perturbation (*γ_r_*(*G*)). As shown in Figure 3, we observed that there are significantly positive relationships between the ratio of coherent FFLs and *γ_r_*(*G*) as for BA and ER model cases (in subfigures (a), (b), (c) and (d), the slopes of the regression lines are 0.66315, 0.36297, 1.38296 and 1.01162, respectively; all P-values<0.0001 using t-test). On the other hand, it is observed that there is little significant relationship between the ratio of coherent FFLs and *γ_r_*(*G*) as for Shuffle I and Shuffle II models (see Figure S1 in File S1). This implies that the relationship between coherent coupling of FFLs and network robustness can depend on the types of random network models.

**Table 2:** Comparison of the ratios of coherent FFLs between each of the real signaling networks (HSN and CCSN) and RBNs.

Network	Maximal Length	Number of Coherent FFLs (A)	Number of Incoherent FFLs (B)	Ratio of coherent FFLs ( 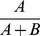 )
HSN	4	125884	131121	0.48981
RBNs (Shuffle I)	4			0.20674±0.00658
RBNs (Shuffle II)	4			0.39719±0.01418
RBNs (BA)	4			0.43721±0.12885
RBNs (ER)	4			0.59210±0.09595
CCSN	6	41961	46676	0.47340
RBNs (Shuffle I)	6			0.14548±0.01004
RBNs (Shuffle II)	6			0.20058±0.01460
RBNs (BA)	6			0.25859±0.11572
RBNs (ER)	6			0.53222±0.09046

### Case study 2: Relationship between coherent feedback loops and network robustness

FBLs actually exist in the form of multiple coupled feedback loops in many biological systems such as budding yeast polarization [43], eukaryotic chemotaxis [44] and Ca^2+^ spikes [45]. For large-scale networks containing a number of coupled feedback loops, the role of feedback loops in realizing the robustness is needed to be fully understood. There was a previous study showing that the coherent coupling of FBLs may be a design principle of a cell signaling network [6]. More specifically, a larger number of coherent coupled FBLs than incoherent coupled FBLs were found in the cell signaling network, and it was argued that such the highly coherent coupling of FBLs strengthens the robustness of the network against state perturbations. However, the effect of coherent FBLs on robustness in a large-scale network was not proven. Hence, we examined it based on the HSN and CCSN. By using the function of FBL search, we determined that both the real networks have a larger number of coherent FBLs than incoherent FBLs as shown in Table 3. To examine whether such the finding in two real networks is a design principle or not, we further conducted two batch-mode simulations on RBNs.

**Table 3:** Comparison of the ratios of coherent FBLs between each of the real signaling networks (HSN and CCSN) and RBNs.

Network	Maximal Length	Number of Coherent FBLs (A)	Number of Incoherent FBLs (B)	Ratio of coherent FBLs ( 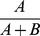 )
HSN	6	7186506	4537973	0.61295
RBNs (Shuffle I)	6			0.50011±0.00016
RBNs (Shuffle II)	6			0.54600±0.01173
RBNs (BA)	6			0.50192±0.00160
RBNs (ER)	6			0.50328±0.03620
CCSN	8	556910	369011	0.60147
RBNs (Shuffle I)	8			0.49999±0.00016
RBNs (Shuffle II)	8			0.50142±0.00156
RBNs (BA)	8			0.50095±0.00179
RBNs (ER)	8			0.50192±0.03864

**Figure 4:**
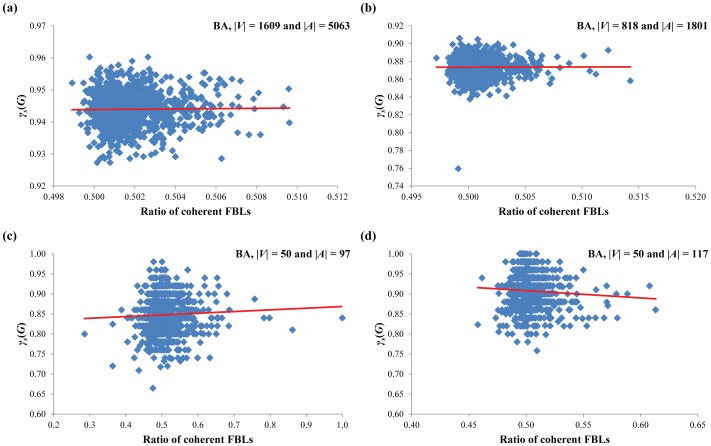
Relationship between the ratio of coherent FBLs and initial-state robustness in Boolean networks by the BA model. (**a**) Result of RBNs of the same size with the HSN. (**b**) Result of RBNs of the same size with the CCSN. (**c**) Result of RBNs with |*V*| = 50 and |*A*| = 97. (**d**) Result of RBNs with |*V*| = 50 and |*A*| = 117. The maximal length of examined FBLs is set to 6, 8, 50 and 12, in (a) through (d), respectively. For robustness against initial-state perturbation, |*S*| is set to 1,024. In (a), (b) and (c), the correlations are not significant (P-value = 0.645, P-value = 0.895 and P-value = 0.167, respectively). On the other hand, the correlation is significantly negative in (d) (P-value = 0.048).

In the first simulation, we tried to verify whether coherent FBLs are ubiquitously found structures in the HSN and CCSN or not. To this end, we generated eight sets of 1,000 random Boolean networks of the same size with each of the real network by using Shuffle I, Shuffle II, BA and ER models, respectively, and examined the ratio of coherent FBLs in them. As shown in Table 3, the ratio of coherent FBLs in the real networks is significantly greater than those of the random networks of all models (using one-sample t-test, all P-values<0.0001). This indicates that coherent FBLs are ubiquitously found in the large-scale signaling networks. In the second simulation, we examined whether the highly coupled structure of FBLs has an effect on the network robustness or not. To this end, we generated two sets of 1,000 random Boolean networks of the same size with the HSN (|*V*| = 1,609 and |*A*| = 5,063) and the CCSN (|*V*| = 818 and |*A*| = 1,801), respectively, by using the BA model and examined the relation of the ratio of coherently coupled FBLs on the robustness against initial-state perturbation (*γ_s_*(*G*)) (Figure 4). As shown in Fig. 4(a) and (b), there was no significant relationship between the ratio of coherent FBLs and *γ_s_*(*G*) (P-value = 0.645 and P-value = 0.895, respectively, using t-test). This implies that the previous hypothesis about the relationship between highly coherent coupling of FBLs and the robustness does not hold in the large scale networks. To examine the dependency of the network size, we additionally generated two sets of 1,000 small-scale random Boolean networks with different network densities: (|*V*| = 50 and |*A*| = 97) and (|*V*| = 50 and |*A*| = 117), respectively, by using the BA model. For the first set, we also observed a non-significant relationship (Fig. 4(c); the slope of the regression line is 0.04192 and the P-value = 0.167 using t-test). On the other hand, there was a significant negative relationship in the latter set (Fig. 4(d); the slope of the regression line is −0.17934 and the P-value = 0.048 using t-test). Moreover, the random networks generated by ER, Shuffle I and Shuffle II models have also shown similar results (see Figure S2 and S3 in File S1). Taken together, we can conclude that coherent FBLs are ubiquitously found in the real signaling networks but there is no significant correlation between coherent couplings of FBLs and robustness against initial-state perturbations unlike the previous studies having shown the positive correlation of them.

## Conclusions

It was very time-consuming to calculate robustness and to examine FBLs/FFLs in large-scale biological networks. In this study, we developed PANET which employs an OpenCL library to perform robustness calculations and to examine FBLs/FFLs in parallel on multi-core CPUs or GPUs. We also implemented a convenient function for batch-mode simulation on a large number of random Boolean networks. We tested our plugin in two case studies based on two large-scale signaling networks and found interesting results regarding relationships between coherently coupled feed-forward/feedback loops and robustness. In addition, we could verify whether or not those findings are consistently conserved in random networks through batch-mode simulations. In particular, we found that coherently coupled FFLs/FBLs are ubiquitously found in the real signaling networks. Moreover, we found that abundant coherent FFLs can improve robustness against update-rule perturbations in some random network models; however, we observed no significant relation between coherent couplings of FBLs and robustness against initial-state perturbations in any random network model. Our Cytoscape plugin is expected to help us to efficiently investigate the relationships between dynamics and structural properties in a large-scale network.

## Supporting Information

File S1 **Supporting information file.** File S1 includes the following: **Text S1.** A brief introduction to OpenCL. **Text S2.** OpenCL-based parallel computation of robustness. **Text S3.** OpenCL-based parallel examination of feedback and feed-forward loops. **Text S4.** Format of an output file by batch-mode simulation on RBNs. **Figure S1.** Relationship between the ratio of coherent FFLs and update-rule robustness in large-scale Boolean networks by Shuffling models. **Figure S2.** Relationship between the ratio of coherent FBLs and initial-state robustness in Boolean networks by the ER model. **Figure S3.** Relationship between the ratio of coherent FBLs and initial-state robustness in Boolean networks by Shuffling models.
(DOCX)
