# Supplementary material for: PANET: A GPU-Based Tool for Fast Parallel Analysis of Robustness Dynamics and Feed-Forward/Feedback Loop Structures in Large-Scale Biological Networks
Source: PLoS One. 2014 Jul 24;9(7):e103010. doi: 10.1371/journal.pone.0103010 (PMC4109960; doi:10.1371/journal.pone.0103010)
Supplement: File S1 — Supporting information file. File S1 includes the following: Text S1. A brief introduction to OpenCL. Text S2. OpenCL-based parallel computation of robustness. Text S3. OpenCL-based parallel examination of feedback and feed-forward loops. Text S4. Format of an output file by batch-mode simulation on RBNs. Figure S1. Relationship between the ratio of coherent FFLs and update-rule robustness in large-scale Boolean networks by Shuffling models. Figure S2. Relationship between the ratio of coherent FBLs and initial-state robustness in Boolean networks by the ER model. Figure S3. Relationship between the ratio of coherent FBLs and initial-state robustness in Boolean networks by Shuffling models. (DOCX) [file pone.0103010.s001.docx]

SUPPLEMENT

**PANET: A GPU-based tool for fast parallel analysis of robustness dynamics and feed-forward/feedback loop structures in large-scale biological networks**

Hung-Cuong Trinh^1^, Duc-Hau Le^2^ and Yung-Keun Kwon^1,^*

^1^School of Electrical Engineering, University of Ulsan, 93 Daehak-ro, Nam-gu, Ulsan 680-749

^2^School of Computer Science and Engineering, Water Resources University, 175 TaySon, Dong Da, Hanoi, Vietnam

**Text S1. A brief introduction to OpenCL**

In this work, we employed an OpenCL library which is designed to run on any available multi-core central processing unit (CPU) or graphics processing unit (GPU) (http://www.khronos.org/opencl/). It utilizes the tremendous computing power of a normal computer by operating the cores of the CPU or hundreds/thousands of cores in the GPU{Elble, 2010 #112@@author-year}{Elble, 2010 #112}. In general, an OpenCL-executable device is divided into one or more compute units (CUs). Each of these is further divided into one or more processing elements (PEs). CUs refer to cores in a multi-core CPU or streaming multiprocessors in a GPU whereas a PE represents a virtual scalar processor, an arithmetic logic unit of a CPU or a scalar processor of a GPU.

In general, an OpenCL application is divided into two parts, the device and the host programs. The device program consists of special functions, called kernels, which are coded with the OpenCL programming language. On the other hand, the host program offers an interface to manage the device execution flow. In other words, the kernel is a basic unit of executable code that can run on GPU or CPU devices whereas the host program takes responsibility for sending kernels to be executed on devices using command queues.

From a logical data-parallelism respect, the host program defines an *N*-dimensional array of work-items (*N* = 1, 2 or 3) in each of which the same kernel is executed. In addition, work-items are grouped into work-groups, and each work-group performs synchronization between work-items by sharing local memory. From the viewpoint of the OpenCL hardware architecture, the work-groups are distributed to CUs and the work-items in a work-group are executed concurrently on PEs of the same CU.

OpenCL defines a hierarchy of different memory types in terms of functionality, size, and speed. The first type of memory is global memory, which has the largest size and the slowest bandwidth. It can be read and written by the host and the OpenCL device, and thus allows intercommunication between the host and the OpenCL device. The second type of memory is constant memory, which is the part of the global memory that remains constant during the execution of a kernel. The third type of memory is the local memory, which is the smallest but the fastest. Each CU has an individual local memory to be shared by the PEs within the CU. It can be used to synchronize between the work-items in the same work-group. The last one is private memory, which is private to a work-item. Variables defined in the private memory of a work-item are not visible to the other work-items. The programmer must choose the most appropriate memory in order to achieve the best possible performance with the available memory bandwidth.

**Text S2. OpenCL-based parallel computation of robustness**

***(a) Pseudo-codes for robustness computation in parallel***

The following figure shows the pseudo-codes of two important functions, *parallel_computing_attractors_for_all_states* and *parallel_computing_attractors_for_all_rules* which can compute the attractors in parallel for all initial states (*S*) and every update rule (*F*), respectively, given a Boolean network. In computing attractors, we used an array *ATT* where each element *ATT*[*s*, *f*] represents an attractor of a network *G*(*V*, *A*) starting from the initial state *s* and the sequence of update rules *f*. The algorithm iteratively computes a state transition until it arrives at a state which has already been visited. We note that the dashed blocks denote kernel codes which are executed *in parallel* on CPUs or GPUs. In other words, the original NetDS serially computed the attractors for a number of initial states or update rules, whereas PANET computes them in parallel by distributing the tested cases to PEs in the OpenCL device.

| **** | **** |
| --- | --- |

By using those functions, we can easily compute not only the robustness of a node against the initial-state perturbation and the update-rule perturbation (*γ_s_*(*v*) and *γ_r_*(*v*), respectively), but also the robustness of a network *G* against the initial-state perturbation and the update-rule perturbation (*γ_s_*(*G*) and *γ_r_*(*G*), respectively) as shown in the following pseudo-codes.

|  |  |
| --- | --- |

**Text S3. OpenCL-based parallel examination of feedback and feed-forward loops**

***(a) A pseudo-code for efficient FBL search in parallel***

The following figure shows the pseudo-code of the ‘searchFBL’ function to search all feedback loops of a maximum length *L* in a given network *G*(*V*, *A*). For each link (*v_i_*, *v_j_*) ∈ *A*, the algorithm starts to search all the FBLs involving the link (*v_i_*, *v_j_*) based on depth-first-search (DFS). We note that the dashed block explains the searching task. It is a kernel code which can be executed in parallel on CPUs or GPUs. In addition, we improved the search speed by avoiding redundant search (* and ** lines in the pseudo-code).

***(b) A pseudo-code for efficient FFL search in parallel***

The following figure shows the pseudo-code of the ‘searchFFL’ function to search all feed-forward loops of a maximum length L in a given network *G(V, A)*. We have a set of source nodes *V_S_* and a set of destination nodes *V_D_* used to find FFLs. For each link (*v_s_, v_j_*) ∈ *A* with *v_s_* ∈ *V_S_*, the algorithm starts to search all the FFLs involving the link (*v_s_, v_j_*) based on depth-first-search. We note that the dashed block explains the searching task. It is a kernel code which can be executed in parallel on CPUs or GPUs.

**Text S4. Format of an output file by batch-mode simulation on RBNs**

After the batch-mode simulation is completed, two resultant files are created: “net_based_result.txt” and “node_based_result.txt”*.* The former and the latter describe network-based and node-based results, respectively.

***(a) Network-based result***

As shown in the figure below, “net_based_result.txt” consists of 11 network-based results with respect to robustness and FFL/FBL structures of RBNs. Each row describes a result of one RBN.

| **Column** | **Name** | **Description** |
| --- | --- | --- |
| 1 | Network ID | The unique identification number of an RBN |
| 2 | No.Nodes | The number of nodes of an RBN |
| 3 | No.Edges | The number of edges of an RBN |
| 4 | sRobustness | The robustness against initial-state perturbation of an RBN |
| 5 | rRobustness | The robustness against update-rule perturbation of an RBN |
| 6 | NuFBL+ | The number of positive FBLs of an RBN |
| 7 | NuFBL- | The number of negative FBLs of an RBN |
| 8 | NuCoFBL | The number of coherently coupled FBLs of an RBN |
| 9 | NuInCoFBL | The number of incoherently coupled FBLs of an RBN |
| 10 | NuCoFFL | The number of coherently coupled FFLs of an RBN |
| 11 | NuInCoFFL | The number of incoherently coupled FFLs of an RBN |

(Column description in “net_based_result.txt”)


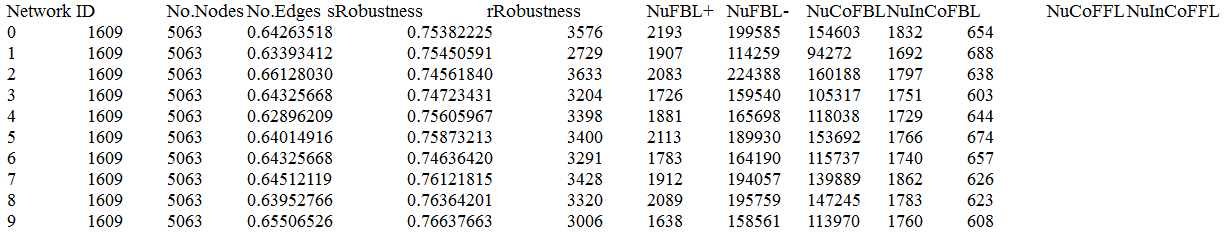


(Example of “net_based_result.txt”)

***(b) Node-based result***

As shown in the figure below, “node_based_result.txt” shows more detailed results than “net_based_result.txt” because it includes the results with respect to robustness and FBL structures at each node level in the RBNs (The result regarding FFL structures are not included for simplicity, though, because there can be as many cases as the number of all pairs of nodes).

| **Column** | **Name** | **Description** |
| --- | --- | --- |
| 1 | Network ID | The unique identification number of an RBN |
| 2 | No.Nodes | The number of nodes of an RBN |
| 3 | No.Edges | The number of edges of an RBN |
| 4 | Node ID | The unique identification number of a node |
| 5 | sRobustness | The robustness against initial-state perturbation of a node |
| 6 | rRobustness | The robustness against update-rule perturbation of a node |
| 7 | NuFBL<=*L* | The number of FBLs whose length <= *L* involved by a node |
|  | NuFBL*=L* | The number of FBLs whose length = *L* involved by a node |
| 8 | PosNuFBL<=*L* | The number of positive FBLs whose length <= *L* involved by a node |
|  | PosNuFBL=*L* | The number of positive FBLs whose length = *L* involved by a node |
| 9 | NegNuFBL<=*L* | The number of negative FBLs whose length <= *L* involved by a node |
|  | NegNuFBL=*L* | The number of negative FBLs whose length = *L* involved by a node |

(Column description in “node_based_result.txt”)


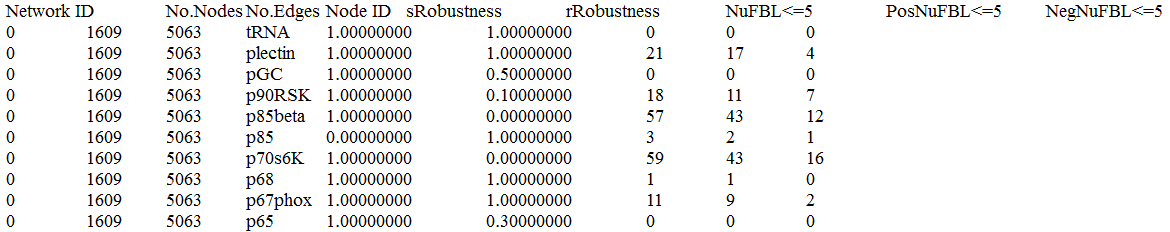


(Example of “node_based_result.txt”)

**(a)**

**(b)**

**(c)**

**(d)**

**Figure S1. Relationship between the ratio of coherent FFLs and update-rule robustness in large-scale Boolean networks by Shuffling models.**

**(a)** Result of Shuffle I-based RBNs of the same size with HSN. **(b)** Result of Shuffle I-based RBNs of the same size with CCSN. **(c)** Result of Shuffle II-based RBNs of the same size with HSN. **(d)** Result of Shuffle II-based RBNs of the same size with CCSN. The maximal length of examined FFLs is set to 4 or 6 for (a) and (c), or (b) and (d), respectively. For robustness against update-rule perturbation, |*S*| is set to 1,024. In (a), (b) and (c), the correlations are not statistically significant (P-values = 0.715, 0.490, and 0.832, respectively). On the other hand, the correlation only in (d) is statistically significant (the slope of the regression line = 0.03297, P-value = 0.015).

**(a)**

**(b)**

**(c)**

**(d)**

**Figure S2. Relationship between the ratio of coherent FBLs and initial-state robustness in Boolean networks by the ER model.**

**(a)** Result of RBNs of the same size with the HSN. **(b)** Result of RBNs of the same size with the CCSN. **(c)** Result of RBNs with |*V*| = 50 and |*A*| = 97. **(d)** Result of RBNs with |*V*| = 50 and |*A*| = 117. The maximal length of examined FBLs is set to 6, 8, 50 and 12, in (a) through (d), respectively. For robustness against initial-state perturbation, |*S*| is set to 1,024. In (a), (b), and (d), the correlations are not statistically significant (P-values = 0.052, 0.384, and 0.080, respectively). On the other hand, the correlation is significantly positive in (c) (the slope of the regression line = 0.05610, P-value = 0.012).

**(a)**

**(b)**

**(c)**

**(d)**

**Figure S3. Relationship between the ratio of coherent FBLs and initial-state robustness in Boolean networks by Shuffling models.**

**(a)** Result of Shuffle I-based RBNs of the same size with the HSN. **(b)** Result of Shuffle I-based RBNs of the same size with the CCSN. **(c)** Result of Shuffle II-based RBNs of the same size with the HSN. **(d)** Result of Shuffle II-based RBNs of the same size with the CCSN. The maximal length of examined FBLs is set to 6 or 8 for (a) and (c), or (b) and (d), respectively. For robustness against initial-state perturbation, |*S*| is set to 1,024. In (a), (b), (c) and (d), the correlations are not statistically significant (P-values = 0.603, 0.356, 0.211 and 0.551, respectively).
